# Effects of testosterone treatment on anal sphincter damage repair in ovariectomized rats

**DOI:** 10.55730/1300-0144.5607

**Published:** 2022-11-20

**Authors:** İrem ŞENYUVA, Duygu Baki ACAR, Hasan Hüseyin DEMİREL, Ece TUNÇ

**Affiliations:** 1 Department of Obstetrics and Gynecology, Faculty of Medicine, Uşak University, Uşak, Turkey; 2 Department of Obstetrics and Gynecology, Veterinary Faculty, Afyon Kocatepe University, Afyon, Turkey; 3 Bayat Vocational School, Afyon Kocatepe University, Afyonkarahisar, Turkey; 4 Department of Obstetrics and Gynecology, Veterinary Faculty, Afyon Kocatepe University, Afyon, Turkey

**Keywords:** Anal sphincter, fecal incontinence, menopause, testosterone

## Abstract

**Background/aim:**

Fecal incontinence (FI) generally occurs with anal sphincter damage caused by vaginal delivery in women, obvious FI can develop in the postmenopausal stage. This pelvic floor dysfunction has no rational medical therapeutic options. We investigated the effect of testosterone treatment on the anal sphincter structure, serum thiol/disulfide levels, uterine tissue, and body composition in female rats in an experimental menopause-FI model.

**Materials and methods:**

The animal experiments were performed between September and November 2020 at Experimental Animal Application and Research Center, Afyon Kocatepe University, Afyonkarahisar, Turkey. Thirty-two female rats were divided into four groups: sham, saline, 10 mg/kg testosterone undecanoate, 100 mg/kg testosterone undecanoate. Except for the sham group, all the other groups underwent ovariectomy (OVE) to create a menopause model. Two weeks after this procedure, the FI model was created under general anesthesia in all rat groups. At the end of the experiment, the rats were placed under general anesthesia, weighed, and euthanized after recording the data. The anal sphincter region and uterine tissue samples were collected for histopathological examinations, and blood samples were collected for total testosterone and thiol/disulfide homeostasis analyses.

**Results:**

An increase in anal sphincter muscles and connective tissue thickness was observed in the testosterone-administered groups (p = 0.001). No difference was detected between the groups in the total thiol, native thiol, and disulfide balance (p = 0.087, p = 0.604, p = 0.092). The testosterone-treated groups did not have severe uterine epithelial degradation, hyperemia, or increased endometrial thickness (p = 0.186, p = 0.222, p = 0.630). The body weight of all rats increased (p < 0.05), but the omental weight did not increase (p = 0.061).

**Conclusion:**

Testosterone treatment increased the anal sphincter muscle and connective tissue thickness without causing any oxidative stress and did not result in a pathological change in the uterine tissue and body fat composition.

## 1. Introduction

Fecal incontinence (FI), the involuntary passage of solid or liquid fecal material, is a pelvic floor dysfunction that affects women’s social, physical, emotional, and sexual lives [1–3]. The incidence in women is 2%–25%, and it increases with age [4–5]. Damage to and impaired innervation of the muscles in the anal sphincter complex are the most important causes of this dysfunction [6]. Anal sphincter damage due to vaginal delivery results in obvious FI and in occult FI in 0.5%–9.0% and 20%–41%, respectively, of all deliveries, and it may be a factor in FI that occurs in the postmenopausal period [7]. The anal sphincter complex and pelvic floor connective tissue are all susceptible to female sex hormones during the reproductive period [7–9]. Estrogens and progestins improve muscle growth, and estrogens enhance the connective tissue collagen structure [7,10]. However, in the menopausal stage, decreased sex steroids can lead to weaker connective tissue, anal sphincter sclerosis, and decrease muscle quality and mass [5,7,10]. Moreover, postmenopausal estrogen and progestin replacement may trigger FI because of undesirable effects such as degradation of collagen, increased colonic motility, and oxidative stress [10,11].

Would the use of androgens, another type of steroid, be beneficial for treating FI? Pelvic floor muscles and anal sphincter complex are highly sensitive to androgens [8,12]. Androgen receptors (ARs) exist in the anal sphincter complex; the internal anal sphincter (IAS) smooth muscle and connective tissue; and the external anal sphincter (EAS), which is a continuation of the levator ani muscle (i.e. musculus levator ani [MLA]) and connective tissue [8,13]. Androgens stimulate myotubule and protein synthesis and increase lean muscle mass, diameter, and strength; they also have an effect on connective tissue via fibroblasts [14–17]. Androgens’ characteristics are important for muscle strength, healing, and fibrosis [18]. Fibrosis is linked to tissue oxidation and has a deleterious impact on contractility [18]. Clearing free oxygen radicals (e.g., reactive oxygen species [ROS]) that occur during the oxygen consumption in the body through the antioxidation system is an important process against oxidative stress [19]. Testosterone is an anabolic hormone that increases the basal metabolic rate and oxygen consumption, but its effects on oxidative stress and antioxidation depend on tissue type [20]. However, no study exists on the anabolic and oxidative effects of androgens on the anal sphincter’s healing in the present literature.

It should be noted that androgen administration will have an impact on body composition and endometrial tissue in females [21,22]. Androgens enhance lean body mass, but visceral obesity is dose-dependent [22]. The aromatase enzyme in the endometrium converts androgens to estrogens, which is important for endometrial malignity [22]. In the existing literature, these impacts have not been well investigated. Thus, the aim of this study was to show (1) the impact of testosterone treatment, which is an androgenic steroid, on anal sphincter muscle healing and (2) its possible endometrial and body composition effects in female rats in an experimental menopause-FI model.

## 2. Materials and methods

Animal experiments were performed between September and November 2020 at Experimental Animal Application and Research Center, Afyon Kocatepe University, Afyonkarahisar, Turkey. All experiments were performed in accordance with the National Guidelines for the Use and Care of Laboratory Animals and the study was granted by the Animal Experiments Local Ethics Committee of Afyon Kocatepe University (Afyonkarahisar, Turkey; decision no. 49533702/213 of 24.02.2020).

### 2.1. Animals and study design

Thirty-two Sprague–Dawley healthy female rats (8–12 weeks old; 200–250 g), were used. The animals were housed in animal shelters and were fed with standard rat chow and tap water in a day/night period of 12 h at a temperature of 21–24 °C. After the 1-week adaptation period, the rats were randomly divided into four groups: sham, saline, 10 mg testosterone, and 100 mg testosterone.

### 2.2. Menopause and the FI model

Except for the sham group, all the other groups underwent ovariectomy (OVE) to create a menopause model. The rats were placed under general anesthesia with the intraperitoneal administration of xylazine (10 mg/kg, Ege Vet, İzmir, Turkey)–ketamine (50 mg/kg, Ege Vet, İzmir, Turkey) for the operation. A dorsal midline incision was created in the lower abdominal region under sterile conditions, and the ovaries were completely excised. In the sham group of the rats, the bilateral uterine horns and ovaries were identified by making an incision from the median line in the abdomen. The ovaries were manipulated but placed in their anatomical position without being removed. The fascia and skin were sutured with USP 3-0 polyglactin [25].

### 2.3. The fecal incontinence model and treatments

**Figure 1:**
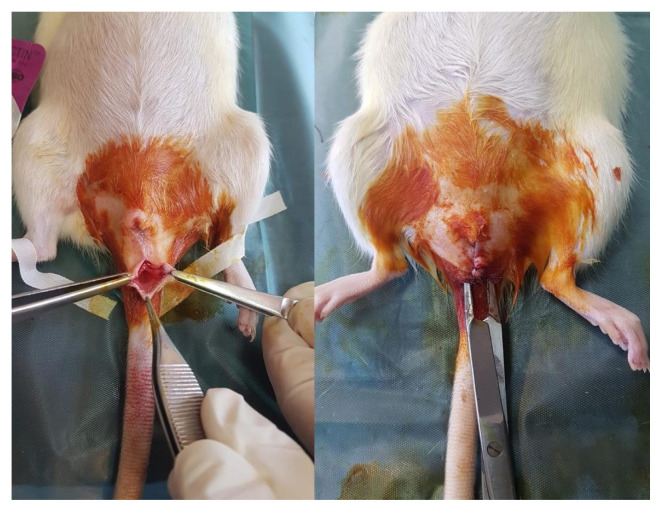
Menopause-fecal incontinence model.

Two weeks after the OVE procedure, the FI model was created under general anesthesia in all rat groups. By modifying the model described by Wai et al. [26], the FI model was achieved with full thickness internal and external sphincterotomy. The rectal mucosa was sutured with 5-0 braided polyglactin in a double layer of primary suturing. Two sutures, set 1 mm apart from each other, were placed on the anal sphincter muscle layer with 5-0 braided polyglactin (Figure 1). All rats were weighed, and data was recorded.

Within the scope of a placebo administration, the saline group received a volume of intramuscular (i.m.) saline injection into the caudal thigh muscle, for once on the day of the FI model, equal to the volume of testosterone hormone injected in the testosterone groups. The rats in the 10 mg testosterone and 100 mg testosterone groups were injected i.m. with, respectively, 10 mg/kg testosterone undecanoate and 100 mg/kg testosterone undecanoate, (Nebido; Bayer Türk Kimya Sanayi Ltd. Şti., İstanbul, Turkey) for once on the day of the FI model [23–25]. The rats in the sham group did not receive any treatment.

### 2.4. Termination of the experimental model

At the end of the experiment (i.e. 3 weeks after the testosterone treatment and saline administration), the rats were placed under general anesthesia, weighed, and euthanized by taking blood from the heart after recording the data. The omentum of each rat was then removed, and the rats were weighed without this part. The anal sphincter region and uterine tissue samples were placed in 10% buffered formaldehyde solution. Histopathological analyses were conducted.

**Figure 2:**
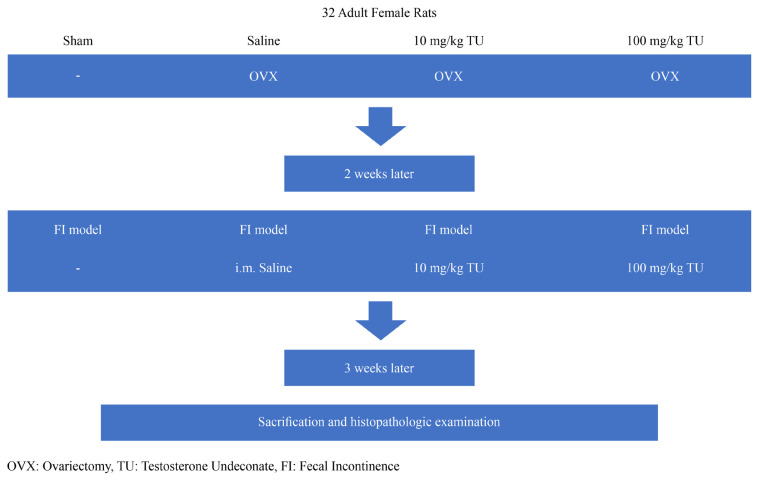
Flow-chart of the experiment.

The blood samples taken from the heart were centrifuged at 3000 rpm for 10 min and their sera were separated. Total testosterone and thiol/disulfide homeostasis were stored at −20 °C until the time of biochemical analysis and thawed at room temperature on the analysis day. The test flow chart is shown in Figure 2. At the end of the experiment (i.e. 3 weeks after the testosterone treatment and saline administration), the rats were placed under general anesthesia, weighed, and euthanized after recording the data. The omentum of each rat was then removed, and the rats were weighed without this part. The anal sphincter region and uterine tissue samples were placed in 10% buffered formaldehyde solution. Histopathological analyses were conducted.

### 2.5. Histopathological examination

**Figure 3:**
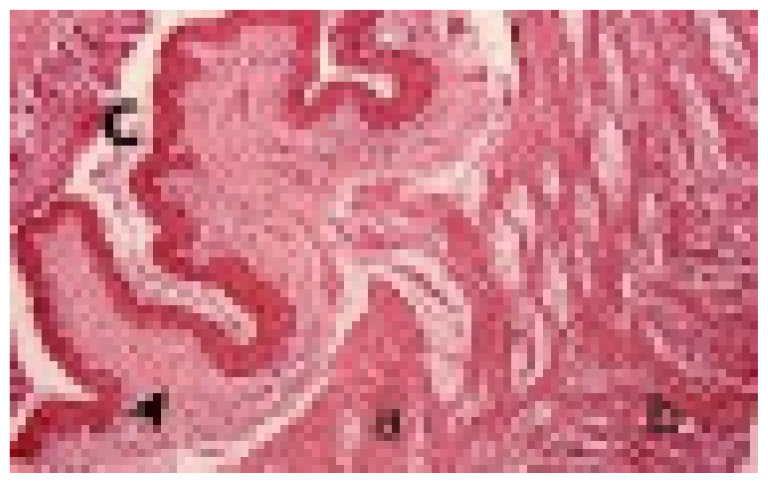
Anal sphincter tissue, a: IAS, b: EAS, c: Anal canal, arrow head: Connective tissue. IAS: Internal Anal Sphincter, EAS: External Anal Sphincter.

Tissue samples for formalin fixation were reduced to 2–3-mm thickness and appropriate sizes and were taken into tissue follow-up cassettes. After being washed in tap water overnight, they were maintained in 50%, 70%, 80%, 96% ethanol and xylol, paraffin with xylol, paraffin melted at 56–58 °C for 2 h for each procedure, and blocked-in paraffin. Samples cut with a microtome (RM 2245; Leica Biosystems, Deer Park, IL, USA) in 5-micron thicknesses from each paraffin block were taken to slides by means of a water bath (HI 1210; Leica Biosystems, Germany). They were dried in an oven for 10 min (Heraterm; Thermo Fisher Scientific, Waltham, MA, USA) and made ready for use in the histopathological methods. All sections were passed through absolute 96%, 80%, 70%, and 50% ethanol series and xylol series and stained with hematoxylin-eosin and Masson’s trichrome staining method [27]. The stained preparations were examined under a binocular head light microscope (Eclipse Ci; Nikon, Tokyo, Japan). The transverse widths of the EAS, IAS, connective tissue, and thickness of endometrium structures were measured with a photomicrometer (μm) at 10× magnification from four areas where the muscle fibers and connective tissue of the anal sphincter complex were regular and the obtained values were averaged (Nikon DS FI3; microscopic digital camera systems, NIS-Elements, Tokyo, Japan) (Figure 3). The single-layered columnar epithelial degeneration in the uterine tissue and the degree of hyperemia in the vessels were evaluated as 0 for “none”; + for “mild”; ++ for “moderate”; and +++ for “severe” [28].

### 2.6. Biochemical analysis

Thiol/disulfide homeostasis (μmol/L) was measured using a novel automatic and spectrophotometric method [29] and total testosterone (pg/mL) levels were measured using the competitive inhibition enzyme immunoassay technique (Rel Assay Diagnostics, Şehitkamil/Gaziantep, Turkey) [30]. The serum samples were thawed at room temperature just before biochemical analysis. The Number Cruncher Statistical System (NCSS) 2007 program (Kaysville, UT, USA) was used for the statistical analyses. During the evaluation of the study data, in addition to using descriptive statistical methods (e.g. mean, standard deviation, median, frequency, ratio, minimum, maximum), the data distribution was also evaluated by using the Shapiro–Wilk test. The Kruskal–Wallis test was used to compare the quantitative data of three or more groups that did not have a normal distribution. The Mann–Whitney *U* test was used to compare two groups that did not have a normal distribution. The Wilcoxon test was used for periodic comparisons. Chi square test was used for qualitative comparison. For the comparison of within-group measurements between groups, Spearman’s correlation test was used. Significance was evaluated at the p < 0.01 and p < 0.05 levels.

## 3. Results

### 3.1. Anal sphincter muscles and connective tissue thickness

The IAS thickness was statistically significantly different between the groups (p < 0.01). IAS thickness in the saline group was lower than that of the other groups (p = 0.001). IAS thickness in the sham group was higher than saline group (p = 0.001) and the IAS thickness of the 100 mg testosterone group was higher than that of the sham (p = 0.001) and 10 mg testosterone groups (p = 0.001). IAS thickness in the 10 mg testosterone group was higher than that of the sham group (p = 0.001).

The EAS thicknesses of all groups were significantly different (p < 0.01). The EAS thickness of the sham group was lower than that of the 10 mg (p = 0.001) and 100 mg testosterone groups (p = 0.001), and it was higher than that of the saline group (p = 0.001), saline group’s EAS thickness was lower than that of the other groups (p = 0.001), the EAS thickness of the 10 mg testosterone group was lower than that of the 100 mg testosterone group (p = 0.001).

The connective tissue was thicker in the sham group than in the saline group (p = 0.001), saline group’s connective tissue was thinner than that of the other groups (p = 0.001) and it was thicker in the 10 mg and 100 mg testosterone therapy groups than in the other groups (p = 0.001, p = 0.001).

In the sham group, no statistically significant correlation existed between IAS and connective tissue, EAS and connective tissue thickness (r = 1.000, p > 0.05, *r* = −0.371, p = 0.468, respectively). In the saline group, the correlation between IAS and connective tissue, EAS and connective tissue was not significant (*r =* 0.429, p = 0.397, r = −0.257, p = 0.623, respectively). In the 10 mg testosterone treatment group, a positive and highly significant correlation was noted between IAS and connective tissue thickness (*r =* 0.733, p = 0.016), but EAS was not significantly correlated with connective tissue thickness (*r =* −0.345, p = 0.328). No significant correlation existed between IAS and connective tissue, EAS and connective tissue thickness in the 100 mg testosterone treatment group (*r =* 0.511, p = 0.132, r = 0.139, p = 0.701, respectively).

**Figure 4:**
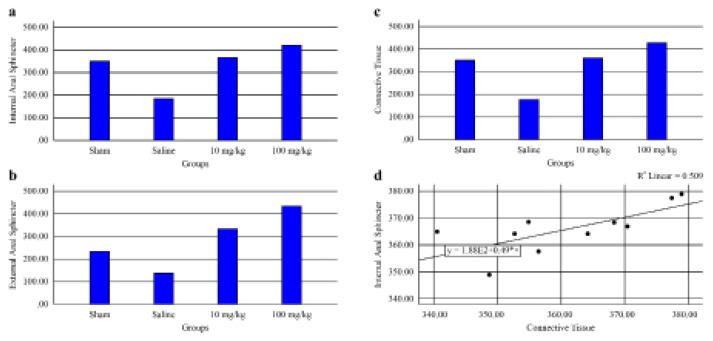
The graphical views of anal sphincter tissue thickness.

The results of IAS, EAS, and connective tissue thickness are shown in Table 1 and Figures 3 and 4.

### 3.2. Testosterone levels and oxidative stress

The blood total testosterone levels showed statistically significant difference among groups (p = 0.013). The sham group values were higher than those of the saline group (p = 0.001), and the 100 mg testosterone group values were higher than those of the saline and 10 mg testosterone group (p = 0.001, p = 0.001). The groups had no statistically significant difference in total thiol level, native thiol level, and disulfide values (p = 0.087, p = 0.604, and p = 0.092, respectively) (Table 2).

### 3.3. Endometrial effect

**Figure 5:**
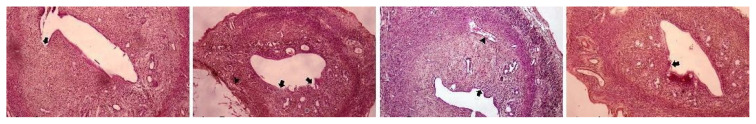
Uterin epithelial degeneration. a: sham, b: saline, c:10 mg testosterone, d:100 mg testosterone. Arrow: Degeneration of single layered columnar epithelium of uterine tissues, Arrowhead: Vascular hyperemia (H&E: Hematoxylin&Eosin).

Table 3 and Figures 5a–5d present the endometrial effect. Degeneration of the single-layered columnar epithelium of uterine tissues and hyperemia in vessels were not statistically significant (p = 0.186, p = 0.222). The testosterone treatment groups did not have severe degenerative effects. No significant changes in endometrial thickness existed between the groups (p = 0.630).

### 3.4. Body and omental weight and abdominal width

The difference between the pre- and poststudy body weight increase in the sham, saline, 10 mg testosterone, and 100 mg testosterone groups was statistically significant (p = 0.027, p = 0.027, p = 0.004, and p = 0.003, respectively) (Table 4). The posttreatment abdominal width and omentum weight values of the groups were not statistically significant (p = 0.092 and p = 0.061, respectively). Table 5 presents omental weight and abdominal width of the groups. No variations in rat feces occurred among the four groups.

## 4. Discussion

Our study demonstrated that testosterone treatment increased IAS and EAS muscle thickness and connective tissue, did not disrupt the thiol-disulfide balance, which is an indicator of oxidative stress, did not cause severe degeneration or pathological changes in the uterine tissue, and did not increase endometrial thickness.

Androgen response in the pelvic floor muscle depends on androgen expression [9]. At birth, the pelvic floor musculature in female rats exhibits a male nature. This morphology disappears when androgen is not in the environment or when it is not taken exogenously [12]. Castration similarly reduces ARs. When exogenous testosterone is administered to castrated rats, myofiber and the satellite cells of their pelvic floor muscles increase [9]. Androgens cause muscle hypertrophy by transforming pluripotent mesenchymal cells into myogenic cells and by increasing protein synthesis in muscles [9,31]. The MLA, which is a main pelvic floor muscle, has generally been studied to demonstrate these androgenic effects. When the structure of the anal sphincter is examined, ARs are more intense than estrogen and progesterone receptors in that location [8]. While the IAS muscle and connective tissue undergo an androgenic effect directly, the EAS striated muscle receives this effect via the connective tissue. [8]. When muscle injury occurs, testosterone treatment increases the satellite cell number, myogenesis stimulation, and muscular growth [32]. In a rat MLA in vivo and in vitro investigation, testosterone therapy increased satellite cell replication [32]. In our experiment, we investigated the anal sphincter region, and we found that testosterone treatment had an anabolic effect on the anal sphincter complex. This finding may be important in the treatment of postmenopausal FI because EAS striated muscle provides the control of fecal urgency, and the IAS is responsible for 70%–85% of the resting sphincter tone [13]. We also found increased connective tissue thickness in the testosterone treatment groups and a positive correlation between IAS muscle and connective tissue only in the low-dose group. This finding may be explained by dose-dependent receptivity. In the literature, some studies [33] have demonstrated that AR expression is suppressed by androgens in particular cell types, thereby limiting the androgen response.

The presence of a thicker connective tissue has clinical implications. Thicker connective tissue can affect contractility because this is the main function of the muscle; in addition, protection from scar tissue in cases of muscle damage is important for optimal muscle recovery [34]. Scar tissue results in incomplete regeneration of the muscle [34]. Transforming growth factor (TGF)-beta is a cytokine involved in fibrosis in muscle tissue and it is involved in the nitric oxide (NO) pathway and androgens regulate monocyte/macrophage production, migration, and function in inflammation [35]. Moreover, androgens have an antifibrotic effect by inhibiting oxidative stress and TGF-beta in striated muscle [31]. As a result, we hypothesized that testosterone’s neutralizing effect on oxidative stress, as well as an increase in the anal sphincter muscle, may have protected the rats against fibrosis. Thus, in the current literature, Oe et al. [36] demonstrated increased EAS muscle mass weight and internal anal pressure with testosterone treatment in an in vivo animal study. They showed positive functional effects of testosterone treatment on the anal sphincter. These findings support our hypothesis.

Testosterone increases the metabolic rate; oxygen consumption; and ROS formation in the testes, muscle, and placenta; and testosterone increases oxidative stress and has an antioxidative effect in prostate and nervous tissue [20]. The thiol-disulfide balance has a critical role in antioxidation [19,20]. Thiol groups combine with ROS in the environment and return to disulfide bond structures, and disulfide is reduced back to thiol groups by oxidant molecules in the environment [19,20]. In oxidative stress, the thiol values are expected to decrease and the disulfide values are expected to increase [16]. An in vivo study [9] demonstrated that androgens increase nitric oxide synthase (NOS) activity and decrease arginase (a powerful antioxidant) activity in the rabbit pelvic tissue. In our experiment, testosterone did not disturb the thiol-disulfide balance. This finding may be related to testosterone increasing the NO synthesis in pelvic tissues.

The anabolic effect of androgens is dose- and plasma concentration-dependent, although the clinical effect is tissue-specific and not correlated with the serum level [9,22]. In humans, testosterone at higher-than-physiological concentrations stimulates myogenesis and provides a strong anabolic effect [9,32]. In three studies using female rats [16,37,38], testosterone treatments applied on the pelvic floor muscle at doses of 0.1 mg, 0.25 mg, and 100 mg/kg, respectively, had effects such as cell proliferation and an increase in fiber number. However, no study has compared dose-dependent effects on the pelvic floor. In our study, although the EAS and IAS thickness occurred with testosterone treatment, the sham and 100 mg groups had similar serum testosterone levels, whereas the saline group had higher serum testosterone levels than the 10 mg group did. This finding showed that the clinical effect was not correlated with the serum level, and that the effect on the tissue is of significant importance.

Androgens have a crucial role in the physiology of the endometrium [39,40]. Androgens have an antiinflammatory and antiproliferative effect on the endometrium [41]. Receptors, cell cycle proteins, and genetics have a role in these effects [42]. However, androgens increase NOS activity and NO regulates endometrial function and blood flow [39,40]. NO is associated with antioxidation [18]. In oxidative stress, ROS come into contact with lipids in the cell membrane and cause cell damage and inflammation through lipid peroxidation [32]. Various forms of NOS are released from the reproductive organs, and it is severely decreased in the postmenopausal period [39]. In this experiment, we did not find severe degenerative changes in the uterine epithelium, and we did not observe any proliferative effect on the endometrium in the testosterone treatment groups. This finding could be related to the neutralizing effects of testosterone on the thiol-disulfide balance and oxidative stress and regulatory effects on the endometrial cells.

Testosterone has an anabolic effect on muscle tissue and a dose-dependent effect on intraabdominal and intramuscular adipose tissue [32]. Testosterone increases insulin sensitivity, accelerates lipolysis by inhibiting lipoprotein lipase via androgen receptor, and inhibits fat cell transformation of mesenchymal precursor cells and maintains them in myogenic state [43]. A clinical study [43] demonstrated that testosterone treatment applied at different levels at high doses causes a decrease in total body adipose tissue and an increase in lean muscle mass. In our study, we detected an increase in the weights of all rats after the experiment; however, no difference occurred in omental weight. The weight increase revealed the anabolic effect of testosterone on the muscle. The lack of a difference between omental weights is explained by the dose.

The limitation of the study is that we used 200–250-g female rats, but the first weighing of the rats was three weeks after the study began (first week adaptation and two weeks after OVE), so the weight of the rats changed during this period.

In conclusion, the current literature contains information on the effect of androgens on the fiber diameter, mass, and catabolism of MLA, and the effect on oxidative stress in various tissues outside the anal sphincter region. However, we believe that our study on the anal sphincter’s healing has revealed for the first time that testosterone basically increases the thickness of the anal sphincter muscle and connective tissue, but it does not disturb the thiol/disulfide balance. Furthermore, no pathologic effect on the uterine epithelium and endometrium and no change in body fat composition occurred. Testosterone may have a significant role in the healing of anal sphincter injury, thereby making it a possible target for FI treatment. To appreciate the role of testosterone, physiological studies to determine the functional state of the anal canal are required.

## Figures and Tables

**Table 1:** IAS, EAS, and connective tissue thickness values of the groups.

Groups	Tissue thickness (μm)	p
	**IAS**	0.001
Sham	1317–1489 (1408.5 ± 69.93)	
Saline	653–768 (712.83 ± 51.42)	
10 mg T	1394–1515 (1458.17 ± 51.6)	
100 mg T	1630–1738 (1678 ± 41.51)	
	**EAS**	0.001
Sham	804–998 (942.17 ± 70.72)	
Saline	521–626 (562 ± 48.06)	
10 mg T	1315–1389 (1343.67 ± 27.73)	
100 mg T	1673–1764 (1705.17 ± 37.02)	
	**Connective tissue**	0.001
Sham	329.35–372.26(350.41 ± 19.28)	
Saline	163.27–192.11(179.09 ± 11.93)	
10 mg T	340.54–378.88(361.24 ± 12.62)	
100 mg T	407.71–451.8(426.99 ± 12.7)	

Data are given as min–max (mean ± SD). T: Testosterone, EAS: External Anal Sphincter, IAS: Internal Anal Sphincter.

**Table 2:** Testosterone and thiol-disulfide values of the groups.

Groups	Biochemical values	p
	**Testosterone (pg/mL)**	0.013
Sham	8.17–13.69 (11.06 ± 1.91)	
Saline	5.62–10.22 (7.13 ± 1.58)	
10 mg T	3.14–15.29 (8.29 ± 4.2)	
100 mg T	8.69–17.89 (12.08 ± 2.61)	
	**TTL(μmol/L)**	0.087
Sham	339–581.7 (407.38 ± 92.77)	
Saline	193.5–799.7 (411.48 ± 216)	
10 mg T	318–720 (466.72 ± 126,9)	
100 mg T	191.4–459.6 (315.17 ± 88.32)	
	**NTL(μmol/L)**	0.604
Sham	107.1–450.1 (241.02 ± 134.81)	
Saline	97.1–549.1 (252.38 ± 171.58)	
10 mg T	83.8–450.6 (177.57 ± 101.78)	
100 mg T	101.2–260.9 (155.6 ± 48.79)	
	**Disulfide**	0.092
Sham	32.03–117.9 (83.18 ± 30.51)	
Saline	18–149.3 (79.55 ± 50.46)	
10 mg T	52.5–275.9 (144.57 ± 71.75)	
100 mg T	9.35–159.6 (79.79 ± 43.65)	

Data are given as min–max (mean ± SD) T: Testosterone, TTL: Total Thiol Level, NTL: Native Thiol Level.

**Table 3:** Uterine tissue histopathological findings and endometrial thickness of the groups.

Histopathological findings	Grade	Sham %	Salin %	10 mg T %	100 mg T %	p

Hyperemia of the vessels	+	16.7	8.3	33.3	41.7	
	++	33.3	33.3	22.7	11.1	0.222
	+++	33.3	66.7	0	0	

Uterine epithelium degenerative changes	+	16.7	8.3	33.3	41.7	
	++	36.4	36.4	18.2	9.1	0.186
	+++	0	100	0	0	

Endometrial thickness (μm)		631.9 ± 32.32 (584.01–665.4)	644.35 ± 26.73 (612.09–679.85)	623.54 ± 31.98 (566.16–658.66)	622.94 ± 35.78 (551.96–670.49)	0.630

Data are given as min–max (mean ± SD), +: mild, ++: moderate, +++: severe, T: Testosterone.

**Table 4:** Body weights of the groups before and after the study.

Groups	Body weight (g)		p
	Before	After	
**Sham**	200–242 (221.83 ± 14.61)	226–261 (247 ± 14.27)	0.027
**Saline**	240–297 (262.83 ± 23.33)	276–366 (306.67 ± 34.91)	0.027
**10 mg T**	235–285 (257.55 ± 14.07)	262–327 (290.36 ± 18.42)	0.004
**100 mg T**	215–290 (259.27 ± 26)	282–336 (310.27 ± 20.58)	0.003

Data are given as min–max (mean ± SD), T: Testosterone.

**Table 5:** Posttreatment abdominal width and omental weights of the groups.

Groups	Posttreatment values	p
	**Abdominal width (cm)**	0.092
Sham	5.6–7.1 (6.4 ± 0.55)	
Saline	6–7.5 (6.72 ± 0.6)	
10 mg T	5.8–6.8 (6.3 ± 0.35)	
100 mg T	6.2–7.4 (6.78 ± 0.42)	
	**Omental weight (g)**	0.061
Sham	2–4 (3.67 ± 0.82)	
Saline	3–12 (7.17 ± 3.66)	
10 mg T	4–9 (5.55 ± 1.51)	
100 mg T	3–7 (5.09 ± 1.38)	

Data are given as min–max (mean ± SD)
